# A Hop Count Based Heuristic Routing Protocol for Mobile Delay Tolerant Networks

**DOI:** 10.1155/2014/603547

**Published:** 2014-06-23

**Authors:** Lei You, Jianbo Li, Changjiang Wei, Chenqu Dai, Jixing Xu, Lejuan Hu

**Affiliations:** Information Engineering College, Qingdao University, Qingdao, Shandong 266071, China

## Abstract

Routing in delay tolerant networks (DTNs) is a challenge since it must handle network partitioning, long delays, and dynamic topology. Meanwhile, routing protocols of the traditional mobile ad hoc networks (MANETs) cannot work well due to the failure of its assumption that most network connections are available. In this paper, we propose a hop count based heuristic routing protocol by utilizing the information carried by the peripatetic packets in the network. A heuristic function is defined to help in making the routing decision. We formally define a custom operation for square matrices so as to transform the heuristic value calculation into matrix manipulation. Finally, the performance of our proposed algorithm is evaluated by the simulation results, which show the advantage of such self-adaptive routing protocol in the diverse circumstance of DTNs.

## 1. Introduction

In traditional data networks such as Internet, there are usually some assumptions of the network model, for example, the existence of at least one end-to-end path between source-destination pair. Any arbitrary link connecting two nodes is assumed to be bidirectional supporting symmetric data rates with low error probability and latency. In addition, the power of each node is considered to be sufficient, thus irrelative to the node throughput. Packets are buffered in intermediate nodes (e.g., routers) and further forwarded to the next-hop relay or successfully received by the destination. In this case, each packet is not expected to occupy the buffer of nodes for a long period of time. However, these all above usually fail in the context of delay/disruption tolerant networks (DTNs), of which the concept was firstly proposed by Kevin Fall in SIGCOMM'03 [1]. DTN architectural designs and explorations dated ever since the first Interplanetary Internet (IPN) project started [2]. Consequently, in the wide variety of work published over the past decade, researchers applied this kind of communication paradigm in different heterogeneous challenged networks, such as mobile wireless sensor networks (MWSNs) [3], mobile ad hoc networks (MANETs) [4], vehicular ad hoc networks (VANETs) [5], and pocket switched networks (PSN) [6]. The deployment and communication in all these networks face diverse challenges, thus called challenged networks by some researchers [1]. In [7], the authors argue that a future Internet architecture should inherently consider challenged networking conditions as a regular case rather than treating them as errors. For example, the difficulty of building sufficient network infrastructures in rural area potentially erodes the progress in universal Internet. Nevertheless, a part of applications addresses the delivery success while having flexible requirement of latency, which is known as “delay-tolerant.” For further popularizing these kinds of applications, we have to reconsider the widely used network architecture so as to relax the assumption of the continuous end-to-end connectivity that is TCP/IP based [8].

**Figure 1:**
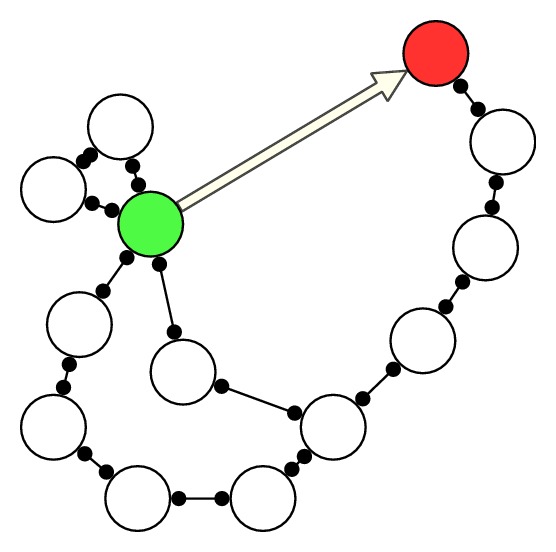
Routing in MANETs.

**Figure 2:**
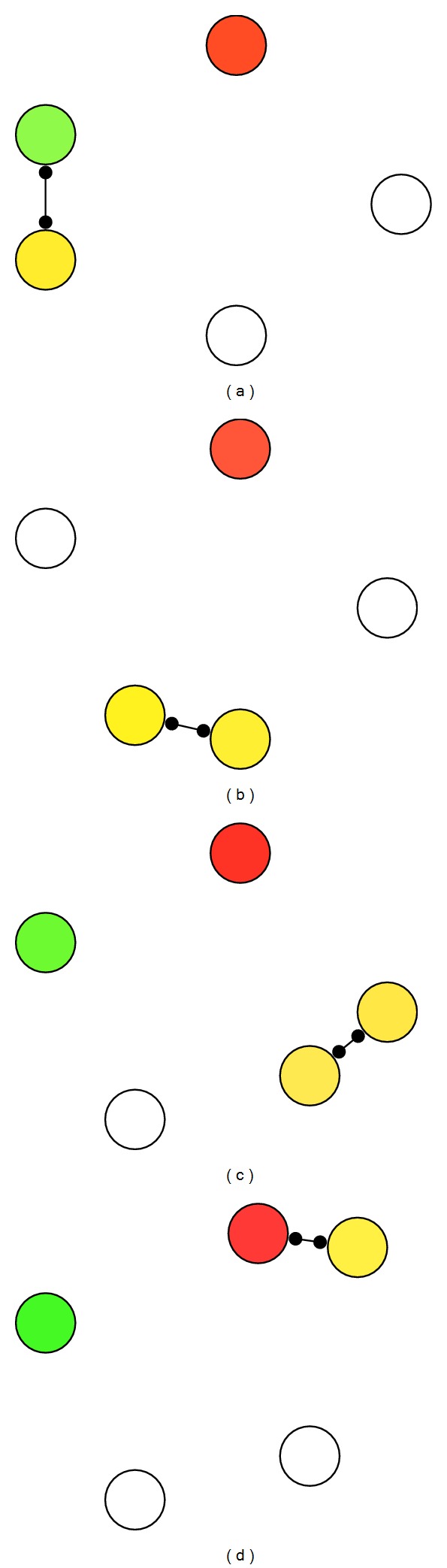
Routing in DTNs.

Since there are some common characteristics between most terrestrial DTNs and mobile ad hoc networks (MANETs), for example, nodes mobility, many research works about routing in DTNs aim to solve the newly arisen difficulties in MANETs that address the “delay-tolerant” property. However, the communication paradigms are different between MANETs and DTNs. As shown in Figure 1, the communication in MANETs usually counts on the possibility of having a prolonged end-to-end connectivity; thus, there might exist an end-to-end path between the source (in green color) and the destination (in red color) in the snapshot MANETs. In a MANET, links among nodes correspond to the connections active at a given instant. Hence, all the information exchanged among nodes passes immediately through this set of active connections only [9]. However, DTNs are characterized by their delay tolerance property, meaning that some content, which has been exchanged among two nodes at a given time, can be exploited and relayed to other nodes in a “store-carry-forward” manner, as illustrated in Figure 2.

In this paper, we propose a hop count based heuristic (HCH) routing scheme for opportunistic networks. In summary, the paper makes the following contributions.

- It employs a heuristic function to route packets towards the respective destinations. The heuristic strategy is based on the hop count information collected by nodes during the routing process.
- It formally defines a custom multiply operation for square matrices, thus transforming the heuristic value calculation into matrix manipulation.
- The performance of HCH is evaluated by the simulation results, which show that HCH has a relatively high delivery ratio and low average end-to-end latency, while introducing acceptable overheads into the network.

The rest of this paper is organized as follows.  Section 2 reviews current research achievements.  Section 3 models the network and answers the preliminary questions.  Section 4 gives the detail about the algorithms. In Section 5, we give our routing protocol. In Section 6, we show the simulation result.  Section 7 concludes this paper.

## 2. Related Work

There have been many research achievements for routing in DTNs. Reference [10] proposed Epidemic routing protocol, which makes use of naive replication strategy that lets each node replicate the message to all encountered nodes so as to achieve the maximizing of the delivery probability of each message. However, the buffer size and energy of nodes are limited, thus constraining the practical performance of this high cost routing scheme. In [11], the authors proposed Spray and Wait (S & W) routing which takes the cost of multiple message replicas into consideration and confined the maximum copies and hops of each message. Based on these two classic routing algorithms, many multicopy routing schemes focused on evaluating contacts opportunities among nodes have been proposed.

In [12], a utility function is introduced as the difference between the expected reward and the energy cost which is spent by the relay to sustain forwarding operations. Reference [13] proposes an active congestion control based routing algorithm that pushes the selected message before the congestion happens. Furthermore, [14] proposes a two-level back-pressure with source-routing algorithm (BP + SR), which reduced the number of queues required at each node and reduced the size of the queues, thereby reducing the end-to-end delay. Reference [15] provides a reliable data delivery scheme for mobile sensor networks with an enhanced delaying technique nodes estimate connectivity and expect interencounter time with sink nodes. Connectivity is estimated based on ratio of past and present connections. When the connectivity is unreliable, nodes delay the transmission for the remaining interencounter duration or per-hop lifetime.

In [16], the authors propose a distributed optimal community-aware opportunistic routing (CAOR) algorithm that computes the minimum expected delivery delays of nodes through a reverse Dijkstra algorithm and achieves the optimal opportunistic routing performance. By proposing a home-aware community model, whereby turning an MON into a network that only includes community homes, the computational cost and maintenance cost of contact information are greatly reduced. Reference [17] applies the evolutionary games to noncooperative forwarding control in MDTNs, of which the main focus is on mechanisms to rule the participation of the relays to the delivery of messages in DTNs. Reference [18] presents two multicopy forwarding protocols, called optimal opportunistic forwarding (OOF) and OOF-, which maximize the expected delivery rate and minimize the expected delay, respectively, while requiring that the number of forwarding operations per message does not exceed a certain threshold.

## 3. Network Model

In this network model, we consider that the network is composed of a number of mobile nodes, which communicate with each other in a peer-to-peer manner. The assumptions are listed as follows:

- all nodes are peer-to-peer, and thus, there is no infrastructure to assist routing. In other words, we do not have some router-like devices to help forwarding the packets in the network. All nodes cooperatively form the network in an ad hoc manner and relay messages through multiple hops to the destination;
- all nodes move in an opportunistic way, which means that, in this network model, it is hard to estimate the exact route for a node. This assumption is for the generality of the application for our routing protocol.

The total number of nodes is denoted by *n* = |*V*|. The node set is represented as *V* = {*v*∣*v* ∈ *V*}. For convenience of analysis, we analyze a certain message generated in node *s* and destined for node *d*. The message is denoted by *M*_*k*_(*s*, *d*), where *k* is the identification. hop(*k*) stands for the passed hop count value of *M*_*k*_. We denote $\bar{\text{hop}}(i,j)$ by the average hop count between any pair of nodes *i* and *j*. Besides, each node of the network is given a unique index ID, which is represented by a lowercase in this paper. The mathematical notations are listed in Table 1.

Given this model, we plan to address the following challenges in the following sections: (a) which metric to use so as to reflect the current network circumstance; (b) how to collect the information from the network, thus dynamically calculating the used metric; (c) how to choose the routing decision according to the estimated network circumstance.

## 4. Hop Count Based Heuristic Scheme

In this section, a heuristic scheme is employed to define the utility function for routing. We first discuss how to collect the needed information from the network. Then, based on the collected information, a heuristic function is proposed to help in making the routing decision.

### 4.1. Information Collected

In our proposed algorithm, we use the hop count metric to help in making the routing decision; since that, it is relatively easy to obtain the hop count information. One way to achieve this goal is letting each packet to carry the passed node(s) information. When a packet reaches a node, the node will then get its passed hop count values record. For example, if message *M*_*k*_ is generated in node *s* and goes along with the path *s* → *p* → *q* → *r*, then, from the head of this message, node *r* may know the hop counts between *s* and *r*, *p* and *r*, and *q* and *r*, as 3, 2, and 1, respectively. And if we average the hop counts value for all received messages, then we can get the average hop count between each pair of nodes, for which we resort to a slide-window mechanism. A matrix *A* is used to record the average hop count information of all nodes, and we let each node maintain such a matrix during the routing process.

**Figure 3:**
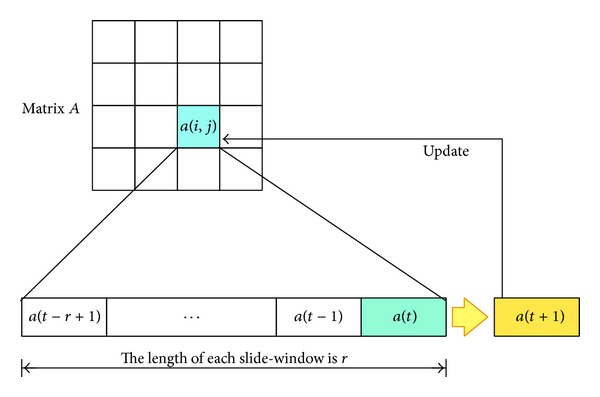
Matrix *A* and the slide-window.

Figure 3 shows the working process of the matrix *A* and its slide-windows. For each element in matrix *A*, there is a corresponding slide-window, which records the historical information about the average hop count carried by the received messages. The length of the slide-window can be appointed by the applications and a longer slide-window indicates a lower node's sensibility to the variation of the network situation. In other words, it is more accurate to reflect the network situation for a longer period of time, and vice versa. By averaging all the values in the slide-window from *t* − *r* + 1 to *t*, we obtain the average hop count value for the next moment *t* + 1.

The process of maintaining the matrix *A* and the slide-windows is illustrated in Algorithm 1. The algorithm requires the information of the arrived packet, denoted by *M*_*k*_, and the next moment, by *t* + 1. The algorithm runs every time when a packet comes. In line 1 we get the ID of the source node for the packet *M*_*k*_. In line 2, all nodes that *M*_*k*_ has passed through are sequentially saved in the array sequence. There are two loops in Algorithm 1, which are responsible for updating the slide-window and the matrix *A*, respectively. By running the first loop, as shown in lines 4–7, the hop count information is saved to the corresponding slot of current time *t* in the slide-window. Lines 8–11 show the second loop that calculates the element values for the matrix *A* by averaging all the records in the slide-window. Thus, each element of matrix *A* reflects the average hop count of a recent period of time between some pair of nodes. In Section 4.2, based on this hop count information, we implement a heuristic metric for routing.

### 4.2. Heuristic Function

Equation (1) shows the heuristic function determined by node *i* and packet *M*_*k*_. hop(*k*) represents the passed hop count of packet *M*_*k*_ and *h*(*i*, *d*) is the heuristic hop count value between the current node *i* and the destination node of *M*_*k*_, denoted by *d*. Thus, the function in (1) is composed of two parts. The first part reflects the actual passed hop count of message *M*_*k*_, while the second part heuristically estimates the prospective required hop count between the current node *i* and the destination node *d*,

(1) $$\begin{array}{c} H\left(i,k\right)=\text{hop}\left(k\right)+h\left(i,d\right). \end{array}$$

Equation (2) exactly shows the heuristic function *h*(*i*, *d*). path_*c*_[*i* → *d*] represents the estimated hop count of the path between nodes *i* and *d*. *m* is the total number of paths between nodes *i* and *d*. Thus, *h*(*i*, *d*) actually stands for the average hop count among all paths between *i* and *d*. We use this value as the heuristic metric for our routing:

(2) $$\begin{array}{c} h\left(i,d\right)=\frac{\sum_{c=1}^{c=m}{\text{path}}_{c}\left[i⟶d\right]}{m}. \end{array}$$

We calculate the heuristic value by introducing a custom operation of matrix ⨀, which is defined as follows.

**Definition 1 .**

Assuming that *M* and *N* are both *n* × *n* matrix, and *O* = *M*⨀*N*, for any element *o*_*i*,*j*_ of the matrix *O*, one has

(3) $$\begin{array}{c} o_{i,j}=\frac{\sum_{k=1}^{k=n}\left(m_{i,k}+n_{k,j}\right)}{w}, \end{array}$$

where

(4) $$\begin{array}{c} w=\left|\left\{c\;|\;c=m_{i,k}+n_{k,j},c>0\right\}\right|. \end{array}$$

Algorithm 2 states the process of heuristic value calculation in detail. The input of this algorithm is matrix *A* maintained by node *i*. By running this algorithm, we finally get all the heuristic value *h*(*i*, ∗) for each message held by node *i*, where ∗ stands for the ID of any possible destination of some packet. The outer loop goes through the set of all the packets of node *i*, as shown in line 1. In lines 2–4, we initialize three necessary variables *h*, *c*, and *M*. These three variables are recursively updated in each iteration of the inner loop. *c* is a local variable and is used to accumulate the total number of paths in each iteration. *M* is initialized to be Λ (the identity matrix) and will be multiplied by *A* in each iteration of the while loop. Lines 5 and 6 obtain the ID of current node and the destination node of *M*_*k*_, respectively. The inner loop ends until the element *m*_*i*,*d*_ of the matrix *M* is zero, which means that there is no *h*-hop path between nodes *i* and *d*. Finally, *h*(*i*, *d*) is set to be the average hop count among all possible paths between the current node *i* and the destination node *d*, as shown in line 13.

**Figure 4:**
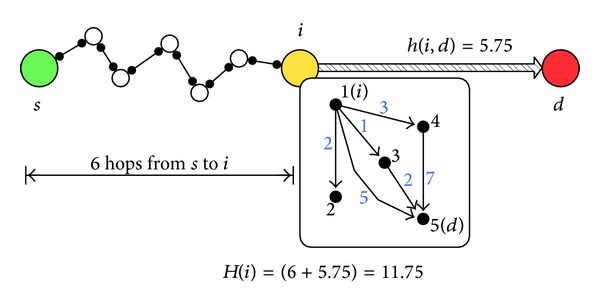
The heuristic value calculation process.

Figure 4 shows an example of the calculation process for the heuristic function. Let us assume that the message *M*_*k*_ is generated at source node *s* and expected to arrive the destination node *d* and node *i* is the current node running the heuristic algorithm. For the packet *M*_*k*_, there have been totally 6 hops from node *s* to node *i*, and thus, we have hop(*k*) = 6. For simplicity, we represent the current node *i* as number 1 and node *d* as number 5, as shown in Figure 4. For the first iteration of the inner while loop, we have

(5) $$\begin{array}{c} M=\Lambda ⨀A=A=\left(\begin{array}{ccccc} 0 & 2 & 1 & 3 & 5\\ 0 & 0 & 0 & 0 & 0\\ 0 & 0 & 0 & 0 & 2\\ 0 & 0 & 0 & 0 & 7\\ 0 & 0 & 0 & 0 & 0 \end{array}\right), \end{array}$$

where we have

(6) $$\begin{array}{c} m_{i,d}=m_{1,5}=5,\\ h=0+5=5,\\ c=0+1=1. \end{array}$$

For the second iteration, we have

(7) $$\begin{array}{c} M=A⨀A=\left(\begin{array}{ccccc} 0 & 0 & 0 & 0 & 6.5\\ 0 & 0 & 0 & 0 & 0\\ 0 & 0 & 0 & 0 & 0\\ 0 & 0 & 0 & 0 & 0\\ 0 & 0 & 0 & 0 & 0 \end{array}\right), \end{array}$$

where we have

(8) $$\begin{array}{c} m_{i,d}=m_{1,5}=\frac{\left[0+0+\left(1+2\right)+\left(3+7\right)+0\right]}{2}=6.5,\\ h=5+6.5=11.5,\\ c=1+1=2. \end{array}$$

The while loop ends before the third iteration, since the element *m*_*i*,*d*_ = *m*_1,5_ = 0, and, finally, we have

(9) $$\begin{array}{c} h\left(i,d\right)=\frac{h}{c}=\frac{11.5}{2}\approx 5.75. \end{array}$$

Notice that the above calculated *h*(*i*, *d*) is an approximate value to its definition in (2). In the example shown in Figure 4, we obtain the average hop count value 6 by using (2), which is close to our calculation result 5.75. Since the ultimate object is to estimate the needed hop count instead of obtaining the accurate average hop count value, it is reasonable to simplify the calculation process with such scheme.

## 5. Routing

In opportunistic routing, packets are relayed in a “store-carry-forward” manner. Due to the lack of continuous end-to-end connectivity, a packet has to be buffered in the relay node for a long period of time. Though multicopy strategy efficiently enhances the routing performance in opportunistic networks, for the network resource is always highly constrained, we should make a trade-off between cost and efficiency.

Table 2 shows the possible choices of two contacting nodes, *v* and *u*. The first principle of our routing is that we do not reduce the number of generated packet replicas. However, the number of replicas is controlled by the second principle that we add new replica to the network only when it is hard for both *v* and *u* to route the packet to the destination. In this case, the multicopy strategy is triggered so as to enhance the routing performance, and otherwise, we choose either *v* or *u* to be the relay node.

Algorithm 3 illustrates the routing decisions in detail. This distributed algorithm runs on each node in the network. As shown in line 1, we firstly get the neighbor set of the current node *v*. Then, in lines 2–16, the routing decision is made upon each packet in node *v* for all its neighbors. The decision is made based on the heuristic function in Section 4.2. Consider the packet *M*_*k*_, of which the source node is *s* and destination node is *d*. In the case that $H(v,k),H(u,k)>\bar{\text{hop}}(s,d)$, for the current node *v* and its neighbor node *u*, the heuristic hop count is larger than the average hop count between *s* and *d*, which indicates that it is hard for both *v* and *u* to route the message to the destination within the average hop count, thus triggering the multicopy strategy.

In lines 6–10, we choose whether to add new replica of *M*_*k*_ according to the variable extra. If extra = true, *v* generates an extra copy of *M*_*k*_ for *u*, as shown in line 12, and this is corresponding to the 1st strategy in Table 2. In lines 13–15, if $H(u,k)\leq \bar{\text{hop}}(s,d)$, *v* then turns over *M*_*k*_ to *u* without keeping the copy in its buffer. In this case, we have $H(u,k)\leq H(v,k)\leq \bar{\text{hop}}(s,d)$; thus, we deem that *u* is capable enough of taking over the message from *v*, corresponding to the 3rd strategy in Table 2. The only remaining case is $H(v,k)\text{leq}H(u,k)\leq \bar{\text{hop}}(s,d)$, which indicates that *u* is not a bit the better choice than *v* and *v* is capable enough to route the packet. Thus, *v* does not forward the packet to *u*, corresponding to the 2nd strategy in Table 2.

## 6. Evaluation

The simulation is evaluated by the Opportunistic Network Environment (ONE) [19]. In detail, we evaluate the Epidemic, binary Spray-and-Wait (S & W), and PRoPHET for performance comparison, using both synthetic mobility model and real trace. The simulation is grouped into the following categories: (1) varying buffer size in Helsinki City Model; (2) varying buffer size in Cambridge-iMote real trace; (3) varying message time-to-live in Helsinki City Model; (4) varying message time-to-live in Cambridge-iMote. The three comparison routing algorithms are listed as follows.

1. *Epidemic*. In this routing scheme, packets received at intermediate nodes are forwarded to all the nodes neighbors (except the one who sends the packet) without employing any flooding control strategy.
2. *S & W (binary edition)*. Spray stage: each node with more than one copy forwards half of the copies to the encountered node with no copy. Wait stage: if the destination is not found in the spray stage, the copy carriers wait for the destination.
3. *PRoPHET*. PRoPHET routing algorithm records history of encounters and transitivity, and the utility metric is based on an encounter probability with the transitivity. PRoPHET estimates a probabilistic metric called delivery predictability, *P*(*a*, *b*), at every node *N*_*a*_, for each known destination *N*_*b*_. This indicates how likely it is that this node will be able to deliver a message to that destination.

We compare the four different routing protocols based on the following criteria.

1. *Delivery Ratio*. Normally, the ultimate goal of routing in DTNs is to achieve great delivery performance. This criterion is the measure of delivery capability for each protocol. When the network resource is sufficient, Epidemic routing usually has the best delivery performance. This is because Epidemic routing always finds the best possible path to the destination. Therefore, it represents the baseline for the best possible delivery performance.
2. *Average Latency*. End-to-end latency is another important concern in DTN routing design. Long average latency means that the message must occupy valuable buffer space for longer, and consequently we desire a low latency value.
3. *Overhead Ratio*. It is desirable to have a low overhead ratio; since that, it reflects the efficiency of message transmission. Overhead ratio is defined to be the number of relay operations (excluding the delivery action) over the number of total delivered messages.

### 6.1. Helsinki City Scenario

**Figure 5:**
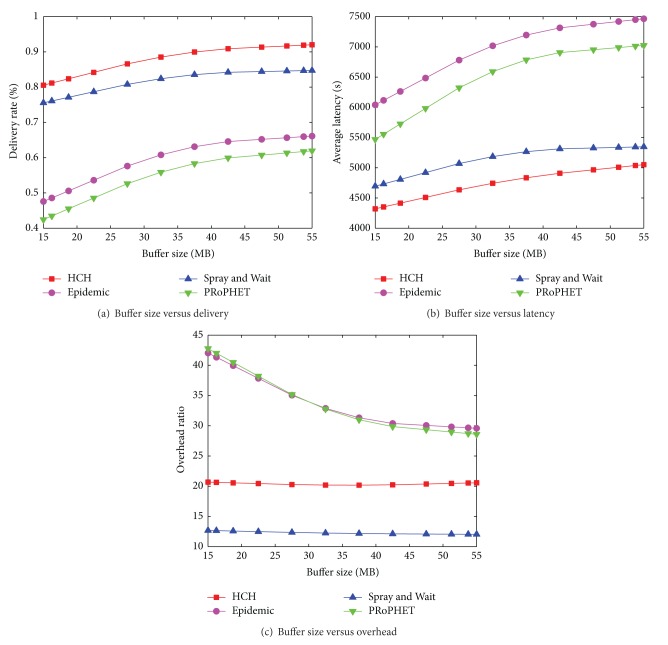
[Helsinki City Scenario] Buffer size versus delivery ratio, average latency, and overhead ratio.

The parameters settings are listed in Table 3. Regarding the results in Figures 5(a), 5(b), and 5(c), our proposed routing protocol achieves the highest delivery ratio and the lowest average latency. The overhead ratio of HCH is higher than S & W and is much lower than that of Epidemic and PRoPHET protocols. The result in Figure 5(a) shows that HCH significantly outperforms Epidemic and PRoPHET and has a slightly higher delivery ratio than S & W. In Figure 5(b), the average latency of HCH is far less than Epidemic and PRoPHET and is slightly lower than S & W. Since HCH heuristically estimates the average hop count thus utilizing the multi-copy strategy in an adaptive way, some unnecessary redundancy is avoided in the network. HCH generates much fewer copies for each message than both Epidemic and PRoPHET. The smaller number of message copies leads to the fewer relay operations, which means the greater efficiency per transmission operation. So HCH has much lower overhead ratio than Epidemic and PRoPHET, as illustrated in Figure 5(c). We can see from Figure 5(c) that the overhead ratio of S & W is lower than HCH. Nevertheless HCH outperforms S & W in both delivery and average latency.

**Figure 6:**
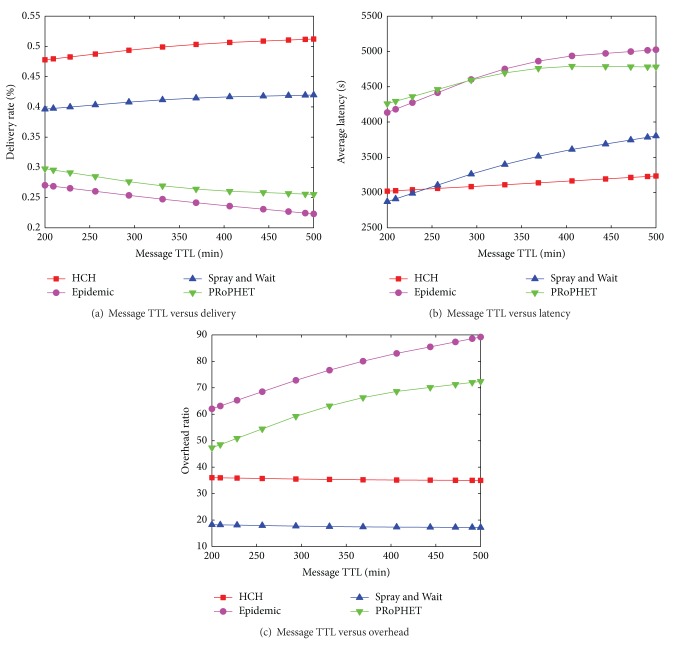
[Helsinki City Scenario] Message TTL versus delivery ratio, average latency, and overhead ratio.

In the simulation of varying message time-to-live, we set the node buffer size (only for cars and pedestrians, not for trams) to be a small value, 15 MB. The result in Figure 6(a) shows that HCH outperforms the other three routing algorithms in message delivery ratio. The average latency of HCH also keeps in the lowest level among all protocols in Figure 6(b). In addition, HCH has good performance in the overhead metric in Figure 6(c). The result in Figure 6(a) shows that when buffer resource is highly constrained, flooding strategy is not a considerable choice for routing. The two flooding based routing algorithms have an unacceptable low delivery ratio, because the buffer resource is scarce thus causing high message dropping probability. HCH performs the best, mostly because the multicopy routing strategy is triggered adaptively according to the current network circumstance. In addition, the average latency of HCH is lower than S & W when message TTL is set to be larger than 250 minutes, as illustrated in Figure 6(b). Finally, from Figures 6(a), 6(b), and 6(c), we know that the message time-to-live property does not have apparent influence on the routing performance.

### 6.2. Cambridge-iMote Trace Set

**Figure 7:**
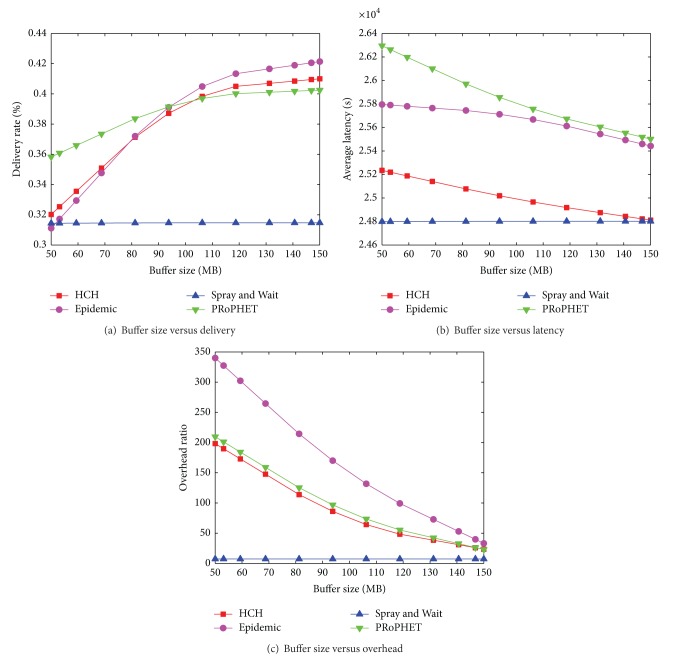
[Cambridge-iMote] Buffer size versus delivery ratio, average latency, and overhead ratio.

The settings of this simulation are listed in Table 4. In this real trace simulation, the buffer size is set to be much larger than that in Helsinki City Scenario.  Figure 7(a) shows that the delivery ratio of HCH approximately equals Epidemic. When the buffer size is larger than 110 MB, HCH wins out PRoPHET in delivery performance. Regarding the result in Figure 7(b), HCH has a higher latency than S & W but is much lower than PRoPHET and Epidemic. The S & W stays in the lowest latency level, while its delivery performance is unacceptable in Figure 7(a). The reason is that the messages in statistics are mainly composed of those that can be delivered quickly. A number of messages that cannot be delivered in a short period are dropped during the routing process. Now we focus on the comparison of the three algorithms, Epidemic, PRoPHET, and our proposed HCH. Though the delivery performance is not evidently better than PRoPHET and is worse than Epidemic, the average latency is much lower than both flooding based protocols. Additionally, in Figure 7(c), Epidemic has the highest overhead ratio, which indicates that much more network resource will be consumed, and consequently the whole lifetime of the network will be short.

**Figure 8:**
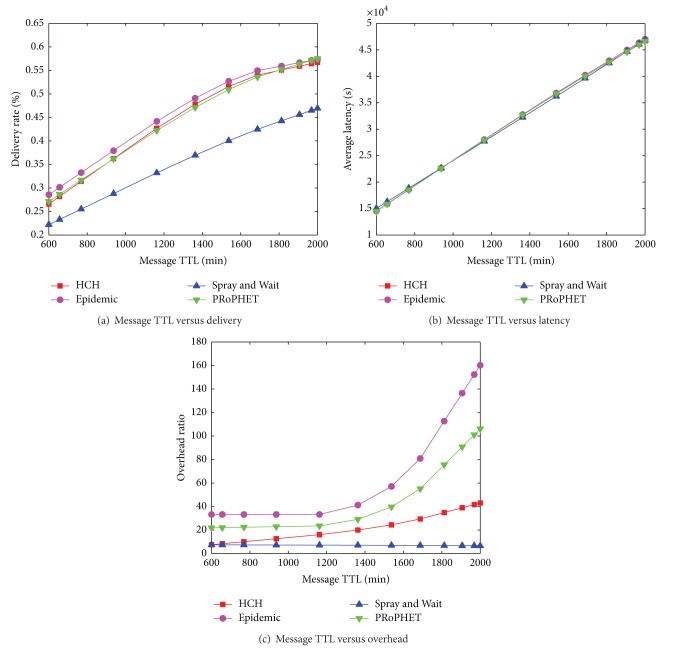
[Cambridge-iMote] Message TTL versus delivery ratio, average latency, and overhead ratio.

In the simulation shown by Figure 8, we set the buffer size to be 100 MB. As shown in Figure 8(a), with the increase of preassigned time-to-live value, the delivery performance of all these algorithms enhances. There are not too much difference among Epidemic, PRoPHET, and HCH. However, as shown in Figure 8(c), the overhead ratio of HCH is only a litter higher than S & W and much lower than both Epidemic and PRoPHET.  Figure 8(b) depicts that all the four algorithms have almost the same performance of average latency. When the message TTL is set to be large, all these algorithms have a relatively high average latency. Nevertheless, the delivery ratio also enhances. However, by comparing Figures 8(a) and 8(b), the latency is rising much quicker than delivery ratio with the increase of message TTL. Thus, we can infer that there are some messages that are hard to be delivered in a short period.

In conclusion, by referring to the Helsinki City Scenario, we know that our proposed HCH has apparent advantage in the performance of average latency and overhead. Besides, HCH is the only one that has good delivery performance in both simulation scenarios among all the four protocols. Thus, we can conclude that the adaptive strategy is very useful so as to pander to the diverse circumstance of DTNs.

## 7. Conclusion

In this paper, we propose a hop count based heuristic routing protocol for mobile DTNs, which makes heuristic estimation based on the hop count information. By employing a slide-window mechanism, we dynamically update the average hop count matrix. Consequently, a heuristic function is defined so as to estimate the prospective required hop count between the current node and the destination node for a packet. A custom operation for square matrices is formally defined, thus transforming the heuristic value calculation into matrix manipulation.

Simulation results show that our proposed HCH outperforms Epidemic, S & W, and PRoPHET in the overall performance of delivery, average latency, and overhead. Due to the diverse circumstance of DTNs, we usually need an adaptive routing algorithm to deal with the frequent changes of network topology. In this case, our proposed HCH is a good choice.

## Figures and Tables

**Algorithm 1:**
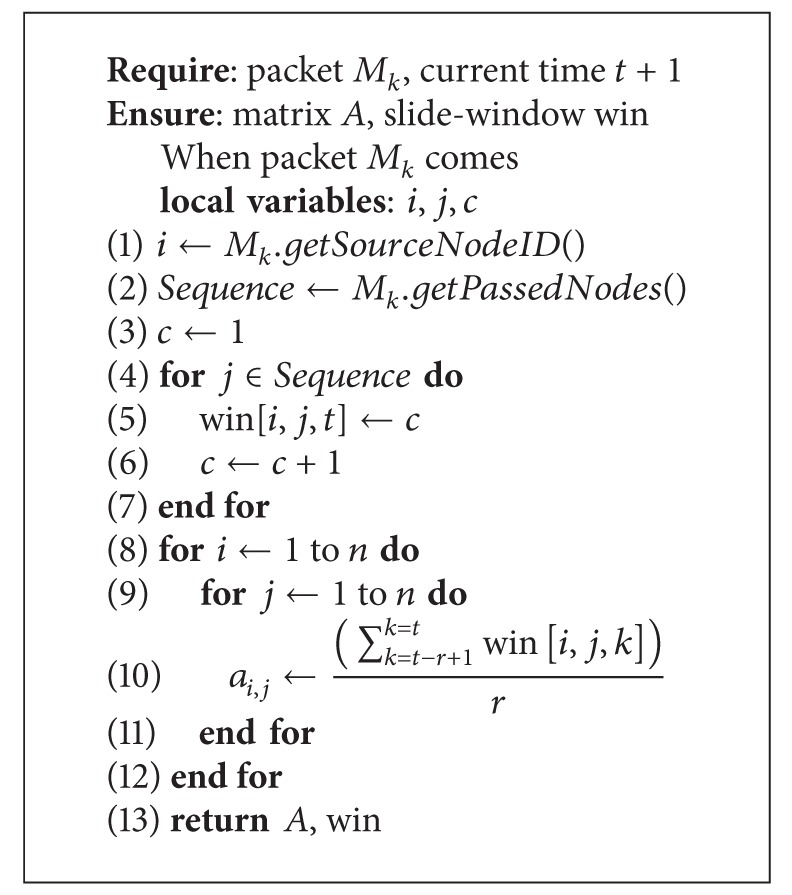
Maintaining the matrix *A* and its slide-windows.

**Algorithm 2:**
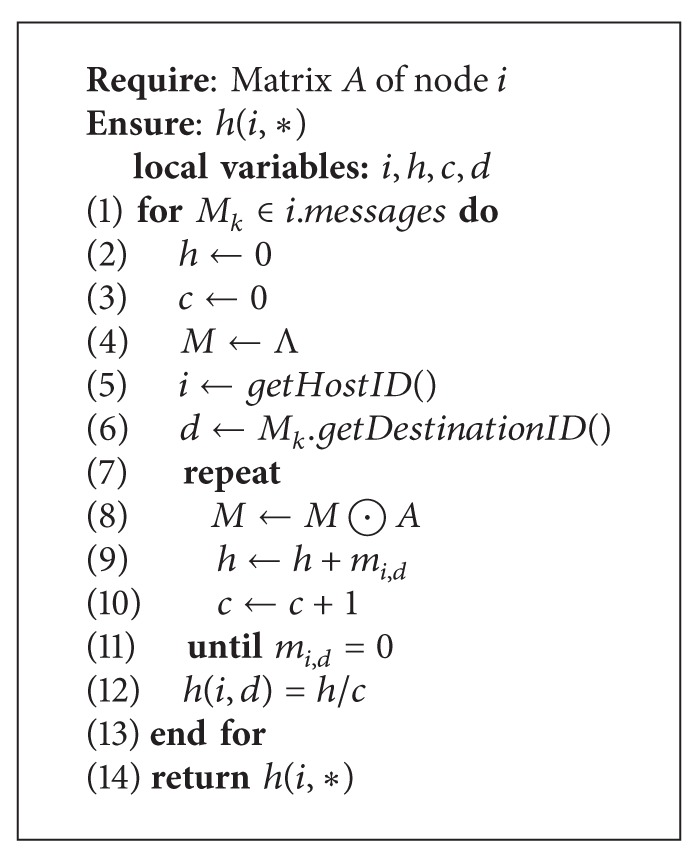
Heuristic value calculation.

**Algorithm 3:**
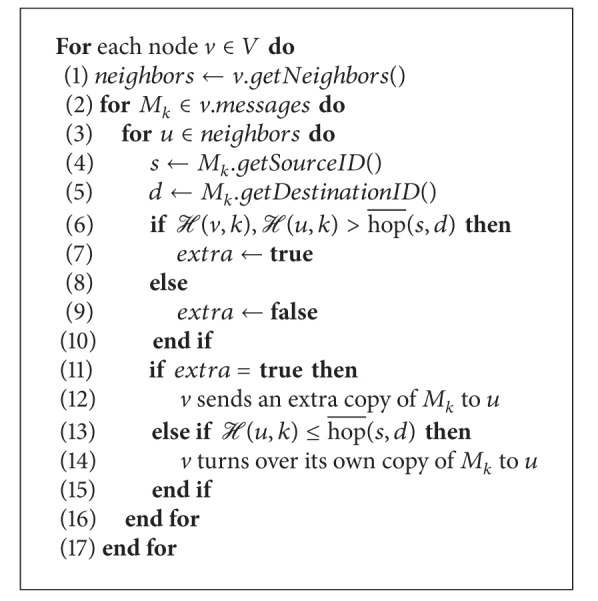
Routing.

**Table 1:** Mathematical notations.

Notation	Meaning
*n*	The total number of nodes
*V*	The set of all nodes in the network
*M*_*k*_(*s*, *d*)	The message identified by *k* with *s* and *d* being the source and destination node, respectively
$\bar{\text{hop}}(i,j)$	The average hop count between any pair of nodes *i* and *j*
hop(*k*)	The passed hop count value of message *M*_*k*_
*h*(*i*, *j*)	The heuristic estimation of the hop count between nodes *i* and *j*

**Table 2:** Optional opportunistic routing decisions.

Strategies	Cases
*v* and *u*	Add an extra message replica in the network
*v*	*u* is not a better choice than *v*
*u*	*u* is a better choice than *v*

**Table 3:** Simulation settings of Helsinki City Scenario.

Parameter name	Range (default value)
Number of nodes	120
World size (m × m)	4500 × 3000
Tickets for S & W	13
Message TTL (min)	200–500 (300)
Simulation time (hours)	12
Message size (KB)	500–1024
Pedestrian buffer (MB)	15–55 (15)
Tram buffer (MB)	500
Bluetooth range (m)	10
High-speed range (m)	1000
Bluetooth bandwidth (KBps)	250
High-speed bandwidth (MBps)	10
Pedestrian speed (m/s)	0.5–1.5
Message interval (s)	35–40

**Table 4:** Simulation settings of Cambridge-iMote trace.

Parameter name	Range
Number of nodes	36
Tickets for S & W	5
Message TTL (min)	600–2000 (1200)
Simulation time (days)	11.5
Message size (KB)	500–1024
Device buffer (MB)	50–150 (100)
Interface bandwidth (KBps)	250
Message interval(s)	35–40
